# COVID-19 Outbreaks at Youth Summer Camps — Louisiana, June–July 2021

**DOI:** 10.15585/mmwr.mm7040e2

**Published:** 2021-10-08

**Authors:** Julius L. Tonzel, Theresa Sokol

**Affiliations:** 1 Louisiana Department of Health, Office of Public Health, Infectious Disease Epidemiology Section.

According to sequencing data reported by CDC, the highly transmissible B.1.617.2 (Delta) variant of SARS-CoV-2, the virus that causes COVID-19, has been the predominant lineage circulating in Louisiana since the week of June 20, 2021 (*1*). In Louisiana, the increased spread of the Delta variant corresponded with the start of the state’s fourth and largest increase in average daily COVID-19 incidence to date (*1*,*2*). This report describes COVID-19 outbreaks in Louisiana youth summer camps as the Delta variant became the predominant lineage during June–July 2021. This activity was reviewed by the Louisiana Department of Health (LDH) and was conducted consistent with applicable state law and LDH policy.*

During June–July 2021, LDH used camp reports and contact tracing data^†^ to identify 28 camp outbreaks^§^ statewide, which included a total of 321 COVID-19 cases^¶^ among an estimated 2,988 campers and staff members. Fourteen (50.0%) of the camps were day camps, and 14 (50.0%) were overnight camps. The mean outbreak size was 11.5 cases (range = 2–59 cases); the mean outbreak size of day camps was 9.3 cases (range = 2–21 cases) and overnight camps was 13.6 cases (range = 2–59 cases). Compared with June–July 2020, when two outbreaks (each with five confirmed camp-associated cases) were identified statewide, this represented a thirty-one-fold increase in confirmed camp-associated cases.

Among the 321 camp-associated cases identified during the June–July 2021 outbreaks, the median age was 12 years (range = 5–54 years), 274 (85.4%) cases occurred among campers (range = 5–18 years), and 47 (14.6%) among staff members (range = 16–54 years). Among all campers with COVID-19, two (0.7%) were fully vaccinated against COVID-19; 133 (48.5%) were age-eligible but not vaccinated (representing 98.5% of the 135 vaccine-eligible campers with COVID-19), and 139 (50.7%) were not age-eligible for vaccination. All cases among staff members occurred in persons who had not received COVID-19 vaccine.

The first 2021 camp outbreak (11 cases) began the week of June 13 (Table). The number of outbreaks and total number of cases peaked during the week of July 4, when nine outbreaks and 118 cases were reported to LDH. The average camp outbreak size peaked at 15.3 cases during the week of July 18. Among the 28 camps with outbreaks during June–July 2021, one (3.6%) required indoor masking for staff members and campers with an outbreak size of eight cases among four staff members and four campers, and one (3.6%) mandated vaccination for all staff members and contractors with an outbreak size of 20 cases among campers. All camps reported some form of cohorting of campers, and seven (25.0%) reported unmasked interactions among cohorts of campers.

**TABLE:** Camp-associated COVID-19 outbreaks and cases reported, by week of symptom onset and percentage of SARS-CoV-2 B.1.617.2 (Delta) variant circulating — Louisiana, June–July 2021

Week beginning	% SARS-CoV-2 Delta variant circulating in Louisiana*^,†^	No. of outbreaks reported	No. of outbreak-associated cases reported (average no. of cases per outbreak)
Jun 1	N/A	0	0 (—)
Jun 6	N/A	0	0 (—)
Jun 13	38.7	1	11 (—)
Jun 20	54.2	3	30 (10.0)
Jun 27	70.1	4	25 (6.3)
Jul 4	81.3	9	118 (13.1)
Jul 11	84.3	4	41 (10.3)
Jul 18	93.5	6	92 (15.3)
Jul 25	96.4	1	4 (—)
**Total**	**—**	**28**	**321^§^ (11.5)**

**Abbreviation:** N/A = not available.

* Variant proportion data not available before June 13, 2021.

^†^ https://covid.cdc.gov/covid-data-tracker/#variant-proportions. Accessed September 26, 2021.

^§^ Median = 35.5; interquartile range = 21.5–98.5.

During the two previously identified camp outbreaks during June–July 2020, COVID-19 vaccines were not available, and all persons aged ≥8 years were required to wear a mask while indoors. However, the statewide mask mandate was lifted on May 25, 2021, ahead of the June–July 2021 camp outbreaks described in this study. LDH recommendations for 2021 youth summer camps were to vaccinate all eligible staff members and campers, incorporate universal indoor masking, and maintain separated cohorts or groups of campers. After the 2021 camp outbreak investigations, because approximately one half of infected campers were not age-eligible for vaccination, LDH reinforced its existing recommendations to vaccinate eligible persons, require masking, and to cohort staff members and campers, and included a new recommendation to test all close contacts of persons with confirmed COVID-19.

This study is subject to at least three limitations. First, genomic sequencing was not performed to genetically characterize SARS-CoV-2 isolates from cases. Second, outbreaks were voluntarily reported to LDH, which might result in underestimation of outbreaks. Finally, the role of differences in camp attendance levels by year on case counts could not be examined.

 The increased number of outbreaks and cases observed in Louisiana youth summer camps in 2021 compared with the previous year coincided with the widespread circulation of the highly transmissible Delta variant. This period also coincided with apparent underutilization of preventive measures such as vaccination, masking, and physical distancing. Multicomponent prevention measures, including vaccination of all eligible adults and adolescents, wearing masks indoors, regular screening testing, physical distancing and cohorting, and increasing ventilation can help prevent transmission of SARS-CoV-2 in settings with youths who cannot be vaccinated (*3*,*4*).

## Footnotes

*
https://ldh.la.gov/assets/oph/Center-PHCH/Center-CH/infectious-epi/Surveillance/sanitarycode_06_21_Revision_final.pdf

† Outbreaks were voluntarily reported to LDH by camp administration or LDH-staffed epidemiologists identified clusters of cases through contact-tracing data.

§ Outbreaks were defined as the occurrence of two or more confirmed COVID-19 cases within a setting, with a date of symptom onset (or date of specimen collection, if asymptomatic) within 14 days of one another.

¶ Cases were defined as positive reverse transcription–polymerase chain reaction or antigen test results for SARS-CoV-2.
